# Molecular and immune correlates of lymphocyte activation gene-3 expression in renal cell carcinoma

**DOI:** 10.1093/oncolo/oyag317

**Published:** 2026-08-11

**Authors:** Harshitha Dudipala, Adam J Dugan, Unnati Jariwala, Karyn Ronski, Jacob Mercer, Eddy Saad, Jad El Masri, Marc Machaalani, Mustafa Saleh, Kayla Viets Layng, Yingying Yu, Sabina Signoretti, David Braun, Sumanta K Pal, Toni K Choueiri, Rana R McKay

**Affiliations:** Moores Cancer Center, University of California San Diego, La Jolla, CA, United States; Tempus AI, Inc., Chicago, IL 60654, United States; Tempus AI, Inc., Chicago, IL 60654, United States; Tempus AI, Inc., Chicago, IL 60654, United States; Tempus AI, Inc., Chicago, IL 60654, United States; Dana Farber Cancer Institute, Boston, MA 02215, United States; Dana Farber Cancer Institute, Boston, MA 02215, United States; Dana Farber Cancer Institute, Boston, MA 02215, United States; Dana Farber Cancer Institute, Boston, MA 02215, United States; Tempus AI, Inc., Chicago, IL 60654, United States; Tempus AI, Inc., Chicago, IL 60654, United States; Dana Farber Cancer Institute, Boston, MA 02215, United States; Yale Cancer Center, New Haven, CT 06510, United States; City of Hope Comprehensive Cancer Center, Duarte, CA 91010, United States; Dana Farber Cancer Institute, Boston, MA 02215, United States; Moores Cancer Center, University of California San Diego, La Jolla, CA, United States

**Keywords:** LAG-3, biomarker, immunotherapy response, renal cell carcinoma

## Abstract

**Background:**

Lymphocyte activation gene-3 (LAG-3) is an immune checkpoint receptor that has emerged as a biomarker of interest but its relationship with outcomes in renal cell carcinoma (RCC) is poorly characterized. This study evaluates molecular and immune correlates of LAG-3 expression and its association with outcomes.

**Methods:**

De-identified DNA-NGS data (xT) and RNA-NGS (xR) from patients (pts) with RCC (*n* = 566) within the Tempus multi-modal database were analyzed using Lens. Eligible pts had first-line (1 L) immunotherapy (IO) and biopsies collected within 1 year of IO. Samples were stratified into 4 quartiles by LAG-3 RNA expression (TPM). Immune cell proportions were estimated from RNA (quanTIseq). Real-world objective response rate (rwORR) was assessed within 90 days of IO. Real-world overall survival (rwOS), defined as time from 1 L IO start to death, lost to follow-up, or 5 years after 1 L, was analyzed using Cox proportional hazard models and *P*-values (Wald test).

**Results:**

The median age was 61 and majority were male (72%), white (79%), with metastases at specimen collection (94%). BAP1 (*P* = .008) and NF2 (*P* = .015) were increased in the highest LAG-3 groups, while VHL, PBMR1, and SETD2 were not. LAG-3 correlated with higher CTLA4, PD1, PD-L1, PD-2, TIM3, and TIGIT RNA expression (all *P* < .001), and increased M1/M2 macrophages, NK, B, CD8 T, and Treg cell % (all *P* < .001). Higher LAG-3 expression was associated with improved rwORR and lower disease progression (*P* = .039). rwOS was not statistically significant (*P* = .2).

**Conclusions:**

LAG-3 is associated with distinct tumor immune features and may serve as a biomarker for early IO response in RCC.

Implications for Practice Our study demonstrates an association between LAG-3 and immune checkpoint gene expression, portraying a tumor immune microenvironment indicative of T-cell dysfunction and adaptive immune resistance. Clinically, in this real-world cohort treated with first-line IO regimens, higher tumor LAG-3 expression was associated with significantly improved response rates and lower rates of progressive disease, suggesting that an inflamed but checkpoint-restrained tumor microenvironment may be more amenable to immune checkpoint blockade. Hence, LAG-3 expression may serve as a clinically informative biomarker of early IO response and help identify patients more likely to derive benefit from first-line IO regimens, potentially guiding treatment intensification strategies.

## Introduction

Renal cell carcinoma (RCC) represents a significant therapeutic challenge in oncology, with approximately 40%-50% of patients with localized disease developing local or distant recurrence despite curative-intent surgery.^1^ The treatment landscape for advanced RCC has evolved significantly since 2018, with immune checkpoint inhibitor combinations becoming the standard of care. While overall survival (OS) for patients with locally advanced/metastatic RCC has improved over the past decade, the majority of patients with metastatic disease succumb to their illness.^2^ Current approved immunotherapy (IO) regimens include dual checkpoint inhibition with nivolumab plus ipilimumab and several checkpoint inhibitors plus VEGF tyrosine kinase inhibitor combinations.^3^^,^^4^

A wide range of biomarkers have been investigated in RCC, such as programmed cell death ligand-1 (PD-L1) expression, genomic alterations, tumor mutational burden (TMB), gene expression signatures, and circulating markers; however, none have been validated for clinical use to guide immunotherapy decisions and identify patients unlikely to benefit from immunotherapy. PD-L1 expression demonstrates limited utility as a negative selection biomarker, given that patients benefit from ICI therapy in both PD-L1-positive and PD-L1-negative tumors in RCC. Although TMB is a marker that correlates with immunotherapy response in cancers such as melanoma and non-small cell lung cancer, RCC is characterized by a relatively low TMB, which limits its predictive value in this disease.^5^ Emerging circulating biomarkers such as kidney injury molecule-1 (KIM-1) have shown early promise, with post hoc analysis of CheckMate 214 demonstrating that early KIM-1 decrease was strongly associated with immunotherapy response but not with sunitinib, though prospective validation is still needed.^6^ Similarly, sMAdCAM-1 serves as a surrogate marker for gut mucosal integrity and has shown prognostic value for both progression-free survival (PFS) and OS. A landmark study by Alves Costa Silva et al. evaluated patients from 3 cohorts (JAVELIN Renal 101, SURF, and NIVOREN) and found that low sMAdCAM-1 was independently associated with worse OS and PFS in the NIVOREN cohort (nivolumab post-TKI) and baseline sMAdCAM-1 > 180 ng/mL was associated with significantly improved PFS and OS in the JAVELIN Renal 101 cohort (avelumab + axitinib vs sunitinib) regardless of treatment arm.^7^ ctDNA-based minimal residual disease assays that have proven useful in other solid tumors remain challenging to implement in RCC due to characteristically low levels of circulating tumor DNA. Therefore, there remains a need to identify biomarkers that help predict treatment response, ultimately improving efficacy while mitigating toxicity of frontline treatment options for metastatic RCC patients.

Lymphocyte Activation Gene-3 (LAG-3) has emerged as a promising immune checkpoint target in RCC. This protein, expressed on T cells, natural killer (NK) cells, B cells, and dendritic cells, can bind HLA class II molecules and plays a role in T cell regulation^8^^,^^9^). In RCC, in vitro studies have shown that programmed cell death 1 (PD-1) blockade increases LAG-3 expression, and concurrent blockade of PD-1 and LAG-3 stimulates interferon gamma production, T-cell infiltration, and checkpoint upregulation.^10^ Thus, LAG-3 expression either alone or combined with PD-1/PD-L1 and other immune exhaustion markers has been linked to an inflamed tumor microenvironment (TME).^8^ Functionally, LAG-3 transmits inhibitory signals through a unique intracellular domain to suppress proximal T-cell receptor (TCR) signaling, and its co-expression with PD-1 on tumor-infiltrating lymphocytes synergistically promotes T cell exhaustion and immune escape^11^^,^^12^). Despite this biological rationale, the utility of LAG-3 expression as a biomarker for immunotherapy response in RCC has not been well characterized. In RCC, there remains a limited understanding of LAG-3 expression patterns and association with outcomes. Therefore, the purpose of this study was to investigate LAG-3 RNA expression as a biomarker in RCC patients receiving frontline immunotherapy and its association with clinical outcomes.

## Methods

### Study cohort

The Tempus Lens multi-modal de-identified database was queried to identify patients with a primary diagnosis of RCC who received first line IO based treatment with either (1) nivolumab + ipilimumab, (2) pembrolizumab + axitinib, (3) nivolumab +cabozantinib, or (4) pembrolizumab + lenvatinib; (*n* = 566). Additional inclusion criteria included (1) tissue samples collected within 365 days before and up to 15 days after starting treatment and (2) samples processed using targeted panel DNA-NGS profiling (xT) and whole-transcriptome RNA sequencing (xR) assays. The Tempus xT assay is a clinically validated 596 or 648 gene NGS panel that detects single-nucleotide variants, copy number variants, indels, and chromosomal rearrangements with high sensitivity and specificity as previously described.^13^^,^^14^ Based on the timing of the samples sent to Tempus, some patient samples were sequenced using more recent assay versions than others. Staging was extracted within 90 days before or after sample collection. For patients with multiple samples the sample closest to, yet preceding, the date of their first treatment initiation was used.

### LAG-3 expression quartiles

RNA-seq data were normalized by computing transcripts per million (TPM) for all protein-coding transcripts, followed by log₂(TPM + 1) transformation. These values were further corrected to account for potential assay and batch effects. Samples were stratified into 4 quartiles by LAG-3 RNA expression (Q1: <1.98 TPM, Q2: 1.98-2.66 TPM, Q3 2.67-3.66 TPM, Q4 > 3.66 TPM).

### Biomarker analysis

#### Immunotherapy markers

PD-L1 status was assessed using the 22C3 clone antibody with standard immunohistochemical protocols performed through Tempus. TMB was measured by counting all non-synonymous missense, nonsense, in-frame insertion/deletion (indels), and frameshift mutations found per tumor that had not been previously described as germline alterations in dbSNP151, Genome Aggregation Database (gnomAD) databases. Deficient mismatch repair/microsatellite instability-high (dMMR/MSI-H) was determined from NGS data by analyzing indels mutations in an optimized number of microsatellite loci depending on the NGS assay utilized (592-gene panel or WES). Loci number analyzed was >2000 for all NGS assays. The threshold to determine MSI-high varied depending on the NGS assay used (592: ≥ 46, which was adjusted to ≥ 21 when the assay was optimized with fewer loci examined; WES ≥ 116). Indeterminate results were reported for samples with low average depth of coverage (<500x for 592 and WES).

#### Immune cell proportions

Relative abundance of immune cell infiltrates in the tumor microenvironment were calculated using quanTIseq (RRID: SCR_022993), which attempts to deconvolute the RNA-seq-based gene expression data via constrained least squares regression. The resulting values are the estimated proportions of 10 different cell types using the TIL10 reference matrix.

#### Somatic DNA alterations

Somatic landscape data were extracted for all patients whose biopsies were assayed with Tempus’ xT panel. Single-nucleotide variants, insertion/deletions, and copy number variations were aggregated to the gene level. Genes were only included in the somatic landscape analysis if variations were detected in at least 2% of the overall sample of patients and they are included on Tempus’ list of clinically reportable genes.

### Outcomes assessed

Treatment response data in the Tempus database were extracted through Tempus’ clinical data abstraction process, a process performed by human abstractors. Treatment response outcomes were assessed from de-identified patient records in the Tempus multimodal database that can be attributed to imaging results, laboratory tests, pathologist evaluation, clinic notes, or physical evaluation. Real world objective response rate (rwORR) was defined as the best response occurring between 15 and 180 days of treatment initiation. Best overall tumor response was categorized as complete response (CR), partial response (PR), stable disease (SD), or progressive disease (PD) based on the best curated response assessments to the 1 L treatment of interest. Patients with CR, PR, or SD were categorized as those with clinical benefit, and PD as those without clinical benefit. Real world OS (rwOS) was defined as the time from first-line immunotherapy start to death or censoring (lost to follow-up or 5-year cutoff). rwOS was analyzed using the Kaplan–Meier curves and Cox proportional hazards models, utilizing risk set adjustment to account for delayed entry. The assumption of independent left truncation was formally tested.

### Statistical analysis

All clinical and molecular characteristics were compared between the LAG-3 expression quartiles. Categorical variables were reported as frequencies and column percentages (%) and *P*-values were calculated using χ^2^ and the Fisher’s exact tests, as appropriate. Continuous variables were reported as medians, first/third quartiles (Q1, Q3), and minimum/maximums (Min–Max.), and *P*-values were calculated using the Kruskal–Wallis Rank Sum tests. Statistical significance was set at *P* ≤ .05 for all 2-sided tests. All analyses were done in R programming language, version 4.4.1 (R Foundation for Statistical Computing, Vienna, Austria). All the Kaplan–Meier plots were generated using the survminer R package, version 0.5.0.^15^ All other figures were generated using the R package ggplot2, version 3.5.2.^16^

## Results

### Baseline characteristics

A total of 566 patients with RCC who received immunotherapy-based first-line therapy were included in the analysis (Table 1). The median age was 61 years and was similar across LAG-3 expression quartiles. Most patients were male (72%), and the gender distribution was consistent across LAG-3 expression quartiles. The majority of patients were White (79%), non-Hispanic (79%) and had no history of smoking (57%). Clear cell RCC was the predominant histological subtype and the histologic distribution across LAG-3 expression quartiles was consistent. The majority of patients (94%) had metastatic disease at the time of biopsy collection. Among metastatic disease patterns, higher LAG-3 expression was associated with a lower incidence of liver metastases (*P* = .019). No significant differences were observed in the distribution of adrenal gland (*P* = .656), bone (*P* = .561), brain (*P* = .337), or lung (*P* = .193) metastases across quartiles. Of the total patients, 285 (50%) had undergone a nephrectomy prior to first-line treatment, with similar distributions across quartiles. The most common first-line regimen was nivolumab plus ipilimumab (*n* = 268, 47%), followed by pembrolizumab plus axitinib (*n* = 178, 31%), nivolumab plus cabozantinib (*n* = 71, 13%), and pembrolizumab plus lenvatinib (*n* = 49, 9%), with similar distribution across LAG-3 expression quartiles.

**Table 1.** Patient baseline characteristics.

	Overall	Stratified by LAG-3 RNA expression				
		<1.98	1.98-2.66	2.67-3.66	3.67+	*P*-value
**Number of patients**	*N* = 566	*N* = 142	*N* = 141	*N* = 141	*N* = 142	
**Age at initial diagnosis (years)**						.571
** Median (Q1, Q3)**	61 (53, 69)	61 (52, 69)	61 (54, 71)	61 (52, 68)	61 (53, 70)	
** Min–Max**	20-85	20-84	32-85	20-85	33-85	
**Age at sample collection (years)**						.522
** Median (Q1, Q3)**	63 (56, 71)	64 (56, 71)	65 (56, 73)	63 (55, 70)	62 (56, 71)	
** Min–Max**	20-88	25-86	36-88	20-87	34-87	
**Sex, *N* (%)**						.244
** Female**	157 (28)	44 (31)	32 (23)	45 (32)	36 (25)	
** Male**	409 (72)	98 (69)	109 (77)	96 (68)	106 (75)	
**Race, *N* (%)**						.097
** White**	331 (79)	85 (79)	72 (73)	95 (83)	79 (80)	
** Black or African American**	34 (8.1)	11 (10)	12 (12)	9 (7.8)	2 (2.0)	
** Asian**	13 (3.1)	1 (0.9)	4 (4.0)	3 (2.6)	5 (5.1)	
** Other race**	42 (10)	10 (9.3)	11 (11)	8 (7.0)	13 (13)	
** Missing observations**	146	35	42	26	43	
**Race, *N* (%)**						.352
** Non-White**	89 (21)	22 (21)	27 (27)	20 (17)	20 (20)	
** White**	331 (79)	85 (79)	72 (73)	95 (83)	79 (80)	
** Missing observations**	146	35	42	26	43	
**Ethnicity, *N* (%)**						.316
** Hispanic or Latino**	65 (21)	19 (23)	11 (14)	21 (26)	14 (19)	
** Not Hispanic or Latino**	245 (79)	62 (77)	65 (86)	60 (74)	58 (81)	
** Missing observations**	256	61	65	60	70	
**Smoking status, *N* (%)**						.572
** No history of smoking**	325 (57)	84 (56)	78 (55)	76 (54)	87 (61)	
** History of smoking**	241 (43)	58 (41)	63 (45)	65 (46)	55 (39)	
** Missing observations**	79	11	23	21	24	
**Histology group, *N* (%)**						.067
** Clear cell RCC**	425 (75)	99 (70)	113 (80)	106 (75)	107 (75)	
** RCC NOS**	98 (17)	27 (19)	15 (11)	27 (19)	29 (20)	
** Variant histology**	43 (7.6)	16 (11)	13 (9.2)	8 (5.7)	6 (4.2)	
**Nephrectomy prior to first-line therapy, *N* (%)**	285 (50)	69 (49)	60 (43)	65 (46)	55 (39)	.096
**Metastatic status at sample collection, *N* (%)**						.949
** Metastatic**	497 (94)	128 (95)	126 (94)	121 (93)	122 (94)	
** Non-metastatic**	32 (6.0)	7 (5.2)	8 (6.0)	9 (6.9)	8 (6.2)	
** Missing observations**	37	7	7	11	12	
**Number of metastatic sites, *N* (%)**						.288
** Unknown**	150 (27)	33 (23)	38 (27)	42 (30)	37 (26)	
** 1**	196 (35)	57 (40)	43 (30)	44 (31)	52 (37)	
** 2**	114 (20)	25 (18)	37 (26)	31 (22)	21 (15)	
** 3+**	106 (19)	27 (19)	23 (16)	24 (17)	32 (23)	
**Metastasis to adrenal gland, *N* (%)**	60 (11)	16 (11)	17 (12)	11 (7.8)	16 (11)	.656
**Metastasis to bone, *N* (%)**	95 (17)	19 (13)	26 (18)	27 (19)	23 (16)	.561
**Metastasis to brain, *N* (%)**	48 (8.5)	12 (8.5)	9 (6.4)	10 (7.1)	17 (12)	.337
**Metastasis to liver, *N* (%)**	68 (12)	26 (18)	19 (13)	13 (9.2)	10 (7.0)	.019
**Metastasis to lung, *N* (%)**	223 (39)	52 (37)	49 (35)	56 (40)	66 (46)	.193
**First-line therapy, *N* (%)**						.285
** Ipilimumab + Nivolumab**	268 (47)	56 (39)	67 (48)	70 (50)	75 (53)	
** Axitinib + Pembrolizumab**	178 (31)	44 (31)	48 (34)	43 (30)	43 (30)	
** Cabozantinib + Nivolumab**	71 (13)	27 (19)	14 (9.9)	16 (11)	14 (9.9)	
** Lenvatinib + Pembrolizumab**	49 (8.7)	15 (11)	12 (8.5)	12 (8.5)	10 (7.0)	
**Sample site, *N* (%)**						.3
** Kidney**	220 (39)	55 (39)	49 (35)	56 (40)	60 (42)	
** Bone**	72 (13)	13 (9.2)	25 (18)	20 (14)	14 (9.9)	
** Lung**	65 (11)	10 (7.0)	19 (13)	19 (13)	17 (12)	
** Soft tissue**	48 (8.5)	16 (11)	8 (5.7)	10 (7.1)	14 (9.9)	
** Liver**	35 (6.2)	14 (9.9)	10 (7.1)	6 (4.3)	5 (3.5)	
** Lymph node**	32 (5.7)	11 (7.7)	9 (6.4)	5 (3.5)	7 (4.9)	
** Endocrine**	28 (4.9)	9 (6.3)	8 (5.7)	6 (4.3)	5 (3.5)	
** Brain**	26 (4.6)	5 (3.5)	5 (3.5)	8 (5.7)	8 (5.6)	
** Other**	24 (4.2)	7 (4.9)	2 (1.4)	8 (5.7)	7 (4.9)	
** Skin**	11 (1.9)	2 (1.4)	4 (2.8)	1 (0.7)	4 (2.8)	
** Gastrointestinal**	5 (0.9)	0 (0)	2 (1.4)	2 (1.4)	1 (0.7)	

Abbreviation: RCC, renal cell carcinoma; NOS, not otherwise specified.

### Immune correlates

#### Checkpoint gene expression

**Figure 1.**
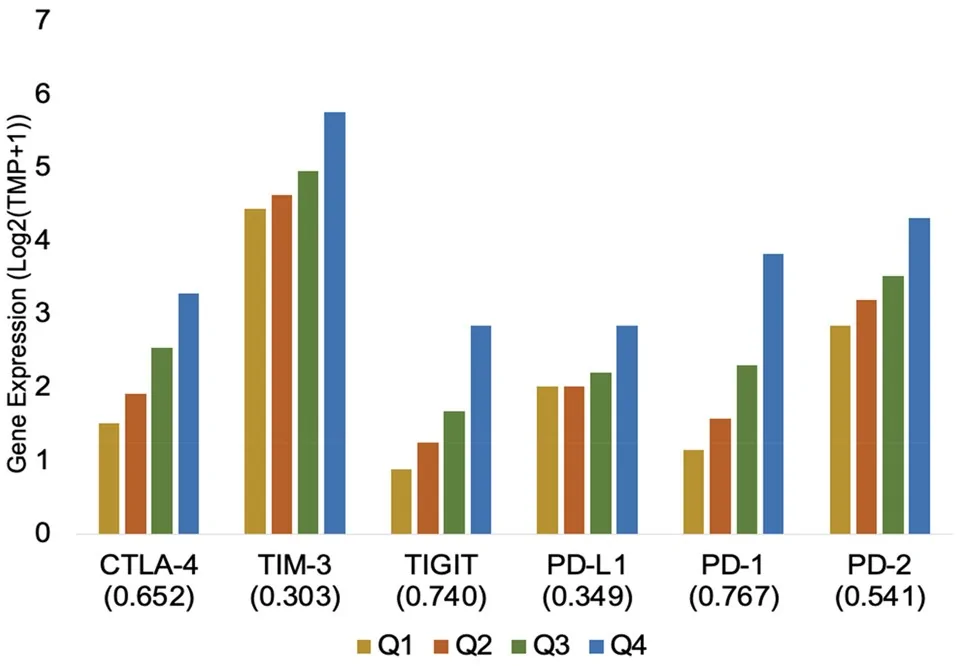
Correlation between checkpoint gene RNA expression and LAG-3 RNA expression quartiles (Pearson coefficient displayed).

Analysis of immune checkpoint gene expression revealed significant positive correlations between LAG-3 RNA expression and co-expression of other immune checkpoint genes (Figure 1, Table S1). PD1 demonstrated the strongest correlation with LAG-3 (ρ = 0.767, *P* < .001), followed by TIGIT (ρ = 0.740, *P* < .001), CTLA-4 (ρ = 0.652, *P* < .001), PD-2 (ρ = 0.541, *P* < .001), CD274/PD-L1 (ρ = 0.349, *P* < .001), and TIM-3 (ρ = 0.303, *P* < .001). Median expression of all checkpoint genes increased progressively across LAG-3 quartiles, suggesting that higher LAG-3 expression is associated with broader immune checkpoint upregulation consistent with an exhausted T cell phenotype within the tumor microenvironment.

#### Immunotherapy markers

**Figure 2.**
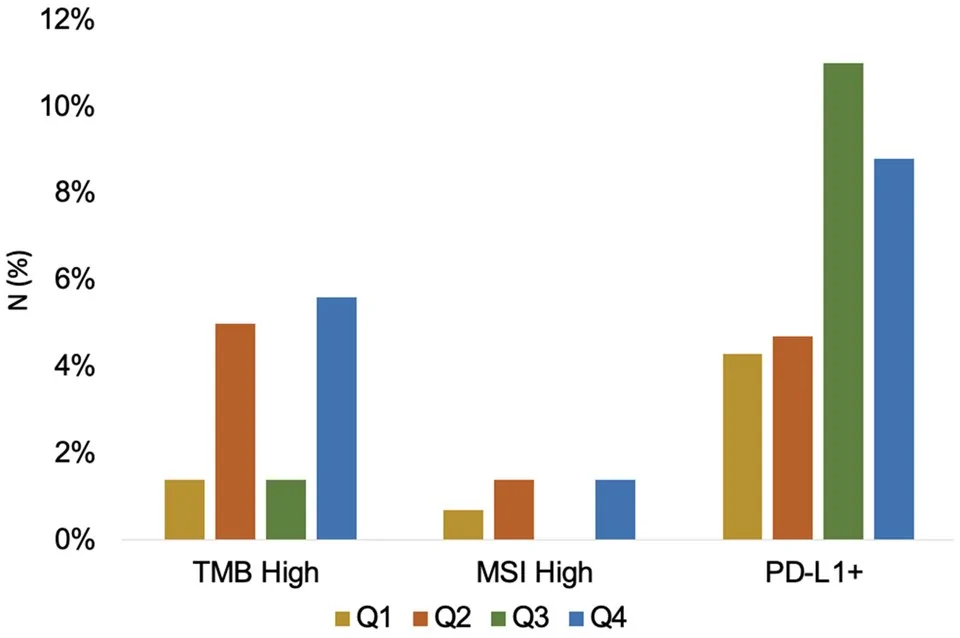
Association between IO markers and LAG-3 RNA expression quartiles.

No significant associations were observed between established immunotherapy biomarkers and LAG-3 RNA expression quartiles (Figure 2 and Table S2). The median TMB was similar across quartiles (3.16 vs 3.68 vs 3.68 vs 3.68 mutations/Mb; *P* = .194), and the proportion of TMB-high (≥10 mutations/Mb) patients was low overall (3.4%) with no significant difference across quartiles (*P* = .084). Similarly, microsatellite instability-high status was rare (0.9%) and did not differ by LAG-3 expression (*P* = .693). Among patients with available PD-L1 data (*n* = 241), PD-L1 positivity by 22C3 was infrequent (7.1%) and was not significantly associated with LAG-3 expression quartiles (*P* = .380). These findings suggest that LAG-3 RNA expression captures a distinct dimension of the tumor immune microenvironment not reflected by conventional immunotherapy biomarkers.

#### Immune cell proportions

**Figure 3.**
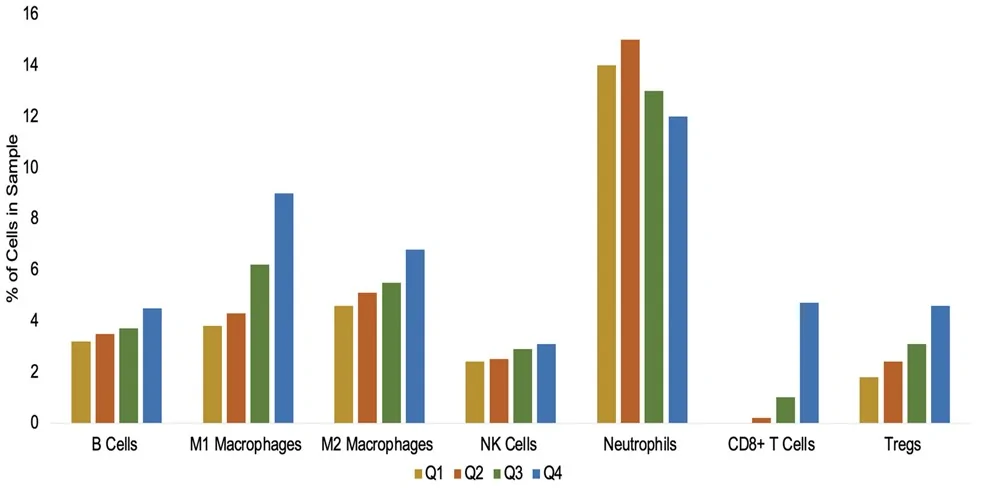
Association between immune cell proportions and LAG-3 RNA expression quartiles.

Analysis of immune cell infiltrate patterns using the quanTIseq algorithm revealed significant associations between immune cell proportions and LAG-3 RNA expression quartiles (Figure 3, Table S3). Higher LAG-3 expression was associated with increased infiltration of cytotoxic effector populations, most notably CD8+ T cells (*P* < .001), as well as NK cells (*P* < .001) and M1-polarized macrophages (*P* < .001). B cell infiltration also increased across quartiles (*P* < .001). Concurrently, immunosuppressive cell populations were also enriched with higher LAG-3 expression, including M2-polarized macrophages (*P* < .001) and regulatory T cells (*P* < .001). Conversely, neutrophil infiltration decreased with increasing LAG-3 expression (*P* = .007). No significant differences were observed for dendritic cells (*P* = .259) or CD4+ T cells (*P* = .145). Together, these findings indicate that higher LAG-3 expression is associated with an inflamed but immune-exhausted tumor microenvironment characterized by both effector and immunosuppressive cell infiltration.

### Somatic DNA associations

**Figure 4.**
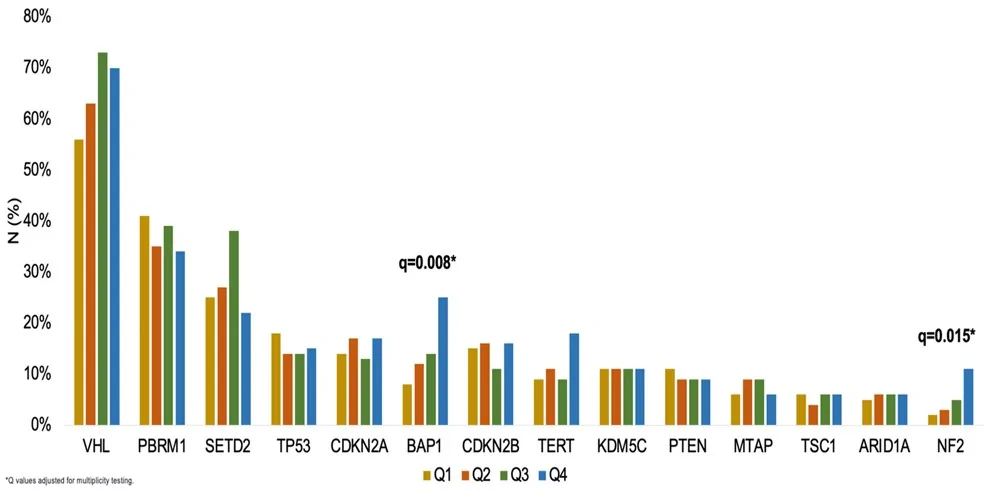
Association between somatic alternations and LAG-3 RNA expression.

Analysis of somatic alterations revealed significant associations between specific gene mutations and LAG-3 RNA expression after false discovery rate correction (Figure 4 and Table S4). BAP1 mutations were significantly enriched in tumors with higher LAG-3 expression, increasing from 7.7% in Q1 to 25% in Q4 (*q* = 0.008). Similarly, NF2 alterations were more frequent in higher LAG-3 quartiles, rising from 2.1% in Q1 to 11% in Q4 (*q* = 0.015). VHL (*P* = .016) and TERT (*P* = .031) mutations showed nominally significant associations with LAG-3 expression but did not retain significance after false discovery rate correction (*q* = 0.075 and *q* = 0.11, respectively). No significant associations were observed between LAG-3 expression and PBRM1, SETD2, TP53, CDKN2A, CDKN2B, KDM5C, PTEN, MTAP, TSC1, or ARID1A mutations (all *q* > 0.9).

### Clinical outcomes

**Figure 5.**
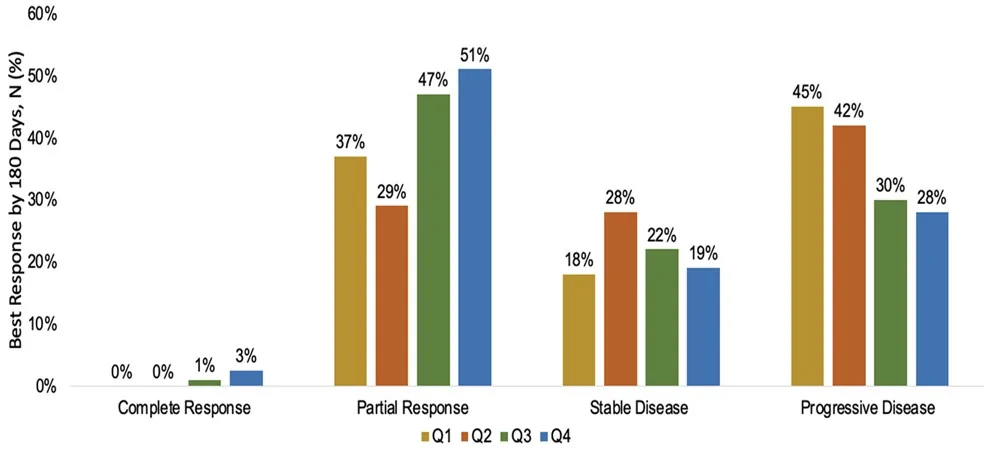
Association between real-world objective response rate and LAG-3 RNA expression.

**Figure 6.**
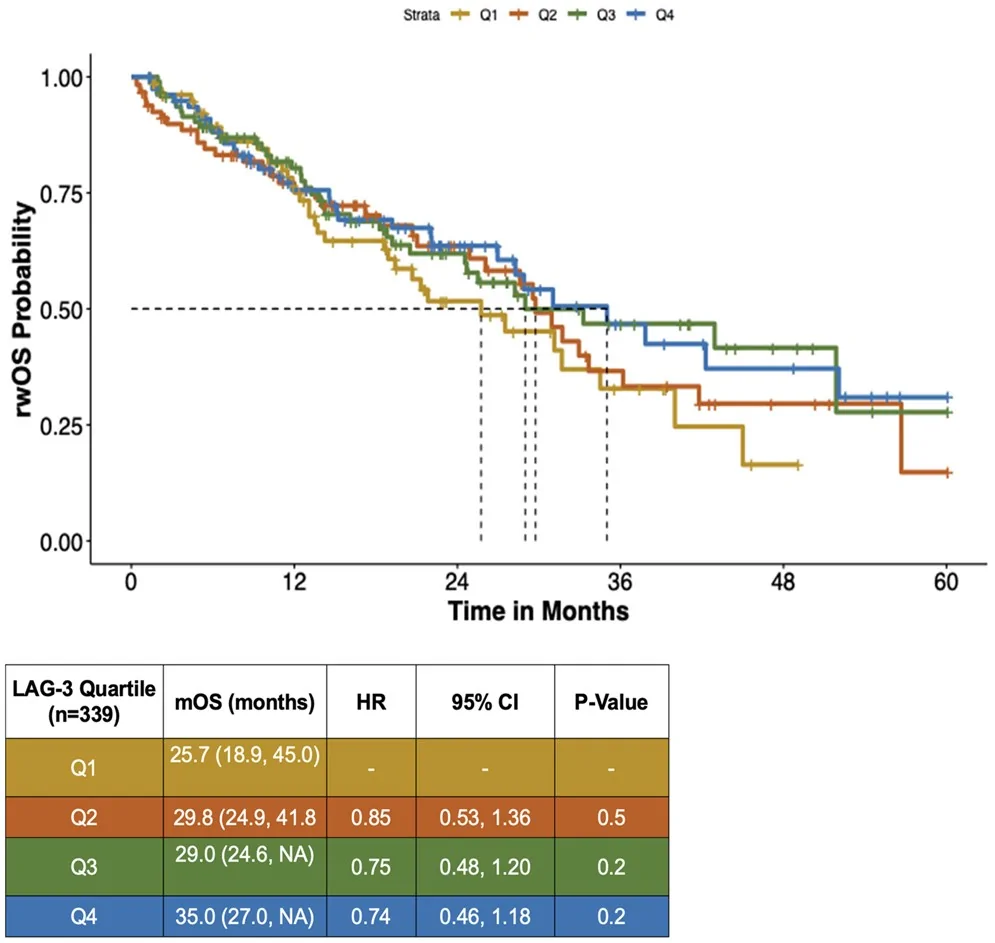
Association between real-world overall survival and LAG-3 RNA expression.

Among patients with evaluable response data at 180 days, higher LAG-3 RNA expression was associated with improved real-world objective response rates (Figure 5, *P* = .039). Patients with clinical benefit (CR + PR + SD) increased from 55% in Q1 to 72% in Q4, while PD rates decreased from 45% in Q1 to 28% in Q4 (*P* = .048). When evaluating rwOS by LAG-3 quartile, there was no statistically significant difference in median OS (Figure 6).

## Discussion

Our analysis identifies elevated LAG-3 expression as a distinct immunobiological biomarker in RCC, correlating with specific metastatic patterns, including lower incidence of liver metastases. High LAG-3 expression was positively associated with other checkpoint gene co-expression and a complex immune landscape characterized by both robust effector infiltration and immunosuppressive signaling. LAG-3 levels were also associated with increased BAP1 and NF2 alterations. Notably, higher LAG-3 expression was linked to improved response rates and lower disease progression, though numerical improvements in OS did not reach statistical significance.

Our study demonstrates an association between LAG-3 and immune checkpoint gene expression, suggesting that higher LAG-3 expression is associated with broader immune checkpoint upregulation consistent with an exhausted T cell phenotype within the tumor microenvironment, indicative of T-cell dysfunction and adaptive immune resistance. The concurrent upregulation of multiple inhibitory receptors, LAG-3, CTLA-4, TIM-3, TIGIT, PD-1, PD-L1, and PD-L2, suggests a terminally exhausted immune state where T cells have encountered persistent antigen stimulation and activated multiple overlapping suppressive pathways.^17–20^ This pattern is characteristic of highly immunogenic tumors that have successfully evaded immune clearance through the coordinated deployment of redundant checkpoint mechanisms. The co-expression of these inhibitory molecules creates a synergistic suppressive network that effectively silences effector T cell function despite the presence of tumor-reactive lymphocytes.^21^ Such extensive checkpoint upregulation typically indicates chronic antigen exposure and represents the endpoint of progressive T cell exhaustion, where cells retain minimal effector capacity.^18^^,^^19^ Therapeutically, this profile presents both opportunity and challenge. While the high checkpoint expression suggests potential responsiveness to immune checkpoint blockade, the redundant suppressive pathways indicate that single-agent checkpoint inhibition may be insufficient.

This microenvironment would likely require multi-targeted combination approaches, potentially including LAG-3, PD-1/PD-L1, and additional checkpoint inhibitors, to effectively restore T cell functionality and achieve meaningful anti-tumor responses. Although sequential blockade could theoretically address compensatory checkpoint activation, salvage CTLA-4 and LAG-3 inhibition after PD-1/PD-L1 exposure has shown limited activity in metastatic RCC, suggesting sequential targeting does not recover the efficacy of concurrent combinations.^22–24^

Associations between LAG-3 and immune cell fractions were observed, exhibiting a complex immunological phenotype characterized by concurrent immune activation and exhaustion pathways. The enhanced infiltration of cytotoxic effector populations, including CD8+ T cells, NK cells, and M1-polarized macrophages, alongside increased B cell presence indicates robust tumor antigen recognition and active cellular and humoral immune responses^25^^,^^26^). However, this pro-inflammatory environment has concomitantly upregulated multiple immunosuppressive mechanisms, including M2 macrophage polarization, regulatory T cell expansion, and elevated LAG-3 checkpoint receptor expression. The decreased neutrophil infiltration may reflect a shift from acute inflammatory responses toward a more chronic, organized immune reaction. This immunological profile is consistent with an adaptive resistance phenotype, where initial immune recognition has triggered compensatory immunosuppressive pathways that establish immune equilibrium rather than tumor clearance.^27^ The high LAG-3 expression, in particular, may suggest active T cell exhaustion and functional impairment of the otherwise robust effector cell infiltrate.^28^ From a therapeutic perspective, this microenvironment represents a rational target for combination checkpoint inhibition strategies, particularly LAG-3 blockade, as the preserved effector cell populations indicate retained immunogenic potential that could be unleashed through strategic immune checkpoint modulation.

LAG-3 expression also correlates with specific somatic DNA alterations, such as BAP1 and NF2. BAP1 is a loss-of-function mutation associated with enhanced tumor immunogenicity through increased neoantigen presentation, upregulated MHC class I expression, and enhanced interferon signaling, promoting T-cell infiltration.^29^^,^^30^ NF2 alterations can dysregulate Hippo pathway signaling and potentially influence immune surveillance mechanisms.^31^ The association suggests that BAP1/NF2-altered tumors may generate persistent antigenic stimulation or immunosuppressive cues, driving adaptive upregulation of inhibitory receptors, ultimately creating an immune-infiltrated microenvironment with features of T-cell exhaustion.^29^ Clinically, this phenotype represents a more biologically aggressive subgroup driven by tumor suppressor loss, thus supporting the rationale for targeting LAG-3–mediated immune suppression in BAP1/NF2-altered tumors. These patients may be more likely to benefit from therapies aimed at reversing T-cell exhaustion (dual checkpoint blockade) while targeting tumor-intrinsic vulnerabilities created by BAP1/NF2 loss. Furthermore, integrating BAP1/NF2 alteration status with LAG-3 expression could serve as a predictive biomarker of response to dual-checkpoint strategies and may ultimately define a subgroup with heightened susceptibility to LAG-3 targeted combination immunotherapy.

Clinically, in this real-world cohort of patients treated with first-line immunotherapy regimens, higher tumor LAG-3 expression was associated with significantly improved response rates and lower rates of progressive disease, suggesting that LAG-3 expression may serve as a clinically informative biomarker of immunotherapy response. Patients in the highest LAG-3 expression quartile experienced the greatest objective response rates, suggesting that an inflamed but checkpoint-restrained tumor microenvironment may be more amenable to immune checkpoint blockade. Hence, stratification of baseline LAG-3 expression could help identify patients more likely to derive early clinical benefit from first-line IO regimens, potentially guiding treatment intensification strategies, such as dual checkpoint blockade. However, there was no association between LAG-3 expression and rwOS, highlighting that early tumor response may not directly translate into survival benefit, thus the role of subsequent lines of therapy, resistance mechanisms, and disease biology remain important factors influencing long-term outcomes. Overall, these results support the incorporation of LAG-3 expression into exploratory biomarker analysis, which could support clinical trial enrichment and stratification strategies. Several RCC clinical trials have tested LAG-3 inhibitors and RNA sequencing has been increasingly performed across clinical trials, providing opportunities for near term validation of these results and hypotheses.

### Limitations

While the use of a large-scale, real-world dataset provides comprehensive analytical advantages, this retrospective approach is nonetheless subject to inherent limitations. There could be selection bias toward patients treated at academic centers who have greater access to and higher utilization of molecular profiling, compared to community practices, and selection toward patients who have more aggressive disease and thus more likely to undergo molecular testing. Based on the timing of the samples sent to Tempus, some patient samples were sequenced using more recent assay versions than others (Table S5). Additionally, our immune biomarker analysis was primarily focused on DNA/RNA due to non-existent IHC data for markers beyond PD-L1. To preserve cohort size, multiple first-line IO-based regimens, which differ in efficacy, mechanism, and toxicity, were included. This introduces treatment heterogeneity and could confound associations between LAG-3 expression and outcomes. This real-world database provides the advantage of analyzing rwORR which was extracted from clinical documentation. However, this is not identical to clinical trial standardized RECIST-based radiographic criteria. Finally, limited follow-up intervals and uncaptured post-progression therapies, may introduce a degree of variability into the rwOS findings.

## Conclusion

LAG-3 is associated with distinct tumor immune features and may serve as a biomarker for early IO response in RCC. High LAG-3 expression identifies RCC tumors with enhanced immunogenicity and greater therapeutic responses despite the presence of adaptive resistance mechanisms. Further evaluations in prospective studies are needed to validate these findings.

## Supplementary Material

oyag317_Supplementary_Data

## Data Availability

The deidentified sequencing data are owned by Tempus and cannot be publicly shared because of the data usage agreement. Qualified researchers can apply for access to these summarized data by contacting Tempus and signing a data usage agreement. Legitimate requests for data sharing will be honored for qualified researchers, on reasonable request, as necessary for conducting methodologically sound research.
