# Supplementary material for: Molecular and immune correlates of lymphocyte activation gene-3 expression in renal cell carcinoma
Source: Oncologist. 2026 Aug 11;31(9):oyag317. doi: 10.1093/oncolo/oyag317 (PMC13537524; doi:10.1093/oncolo/oyag317)
Supplement: Supplementary file 1 [file oyag317_supplementary_data.zip › Supplemental Files.docx]

**Supplemental Tables:**

**Supplement Table 1:** **Checkpoint Gene RNA Expression stratified by LAG-3 RNA Expression Quartiles**

|  | **Stratified by LAG-3 Expression** | | | | |
| --- | --- | --- | --- | --- | --- |
|  | **<1.98** | **1.98-2.66** | **2.67-3.66** | **3.67+** | **Pearson’s Corr. with LAG-3** |
| **Number of Patients** | N=142 | N=141 | N=141 | N=142 |  |
| **CTLA4 Expression (log2(TPM + 1))** |  |  |  |  | ρ=0.652, p<0.001 |
| Median (Q1, Q3) | 1.51 (0.95, 2.09) | 1.91 (1.24, 2.65) | 2.54 (1.87, 3.11) | 3.59 (2.96, 4.22) |  |
| Min. - Max. | 0.25 - 3.71 | 0.21 - 4.29 | 0.48 - 4.99 | 1.18 - 6.18 |  |
| **CD274 Expression (log2(TPM + 1))** |  |  |  |  | ρ=0.349, p<0.001 |
| Median (Q1, Q3) | 2.01 (1.56, 2.34) | 2.02 (1.65, 2.44) | 2.21 (1.74, 2.72) | 2.84 (2.30, 3.39) |  |
| Min. - Max. | 0.39 - 5.43 | 0.71 - 6.23 | 0.66 - 5.37 | 1.11 - 5.64 |  |
| **TIM3 Expression (log2(TPM + 1))** |  |  |  |  | ρ=0.303, p<0.001 |
| Median (Q1, Q3) | 4.44 (3.90, 5.83) | 4.63 (4.24, 5.48) | 4.96 (4.47, 5.49) | 5.76 (5.28, 6.21) |  |
| Min. - Max. | 2.34 - 8.34 | 3.04 - 7.46 | 1.74 - 7.99 | 4.21 - 8.37 |  |
| **TIGIT Expression (log2(TPM + 1))** |  |  |  |  | ρ=0.740, p<0.001 |
| Median (Q1, Q3) | 0.89 (0.66, 1.17) | 1.25 (0.93, 1.60) | 1.68 (1.30, 2.10) | 2.84 (2.29, 3.33) |  |
| Min. - Max. | 0.03 - 3.62 | 0.17 - 2.62 | 0.31 - 3.42 | 0.63 - 4.46 |  |
| **PD-1 Expression (log2(TPM + 1)** |  |  |  |  | ρ=0.767, p<0.001 |
| Median (Q1, Q3) | 1.15 (0.72, 1.54) | 1.58 (1.16, 2.06) | 2.30 (1.89, 2.70) | 3.82 (3.22, 4.26) |  |
| Min. - Max. | 0.00 - 6.47 | 0.10 - 3.53 | 0.39 - 4.13 | 1.44 - 6.09 |  |
| **PD-2 Expression (log2(TPM + 1))** |  |  |  |  | ρ=0.541, p<0.001 |
| Median (Q1, Q3) | 2.84 (2.33, 3.52) | 3.20 (2.69, 3.58) | 3.54 (3.10, 3.94) | 4.31 (3.86, 4.73) |  |
| Min. - Max. | 0.93 - 5.15 | 1.24 - 5.67 | 1.84 - 5.02 | 1.04 - 7.03 |  |

**Supplement Table 2: Immunotherapy Markers Stratified by LAG-3 RNA Expression Quartiles**

|  | **Overall** | **Stratified by LAG-3 Expression** | | | | |
| --- | --- | --- | --- | --- | --- | --- |
|  |  | **<1.98** | **1.98-2.66** | **2.67-3.66** | **3.67+** | **P-value** |
| **Number of Patients** | N=566 | N=142 | N=141 | N=141 | N=142 |  |
| **Tumor Mutational Burden** |  |  |  |  |  | 0.194 |
| Median (Q1, Q3) | 3.68 (2.11, 5.26) | 3.16 (2.11, 4.74) | 3.68 (2.63, 5.26) | 3.68 (2.11, 5.26) | 3.68 (2.29, 5.79) |  |
| Min. - Max. | 0.00 - 45.26 | 0.00 - 13.16 | 0.00 - 30.00 | 0.00 - 10.53 | 0.53 - 45.26 |  |
| Missing Observations | 1 | 1 | 0 | 0 | 0 |  |
| **Tumor Mutational Burden of 10+, N (%)** |  |  |  |  |  | 0.084 |
| Low | 546 (97%) | 139 (99%) | 134 (95%) | 139 (99%) | 134 (94%) |  |
| High | 19 (3.4%) | 2 (1.4%) | 7 (5.0%) | 2 (1.4%) | 8 (5.6%) |  |
| Missing Observations | 1 | 1 | 0 | 0 | 0 |  |
| **Microsatellite Instability, N (%)** |  |  |  |  |  | 0.693 |
| high | 5 (0.9%) | 1 (0.7%) | 2 (1.4%) | 0 (0%) | 2 (1.4%) |  |
| stable | 561 (99%) | 141 (99%) | 139 (99%) | 141 (100%) | 140 (99%) |  |
| **Positive PD-L1 Status (22c3), N (%)** |  |  |  |  |  | 0.380 |
| Negative | 224 (93%) | 67 (96%) | 61 (95%) | 65 (89%) | 31 (91%) |  |
| Positive | 17 (7.1%) | 3 (4.3%) | 3 (4.7%) | 8 (11%) | 3 (8.8%) |  |
| Missing Observations | 325 | 72 | 77 | 68 | 108 |  |

**Supplement Table 3: Immune Cell Infiltration Stratified by LAG-3 RNA Expression Quartiles**

|  | **Overall** | **Stratified by LAG-3 Expression** | | | | |
| --- | --- | --- | --- | --- | --- | --- |
|  |  | **<1.98** | **1.98-2.66** | **2.67-3.66** | **3.67+** | **P-value** |
| **Number of Observations** | N=566 | N=142 | N=141 | N=141 | N=142 |  |
| **Percent of B Cells** |  |  |  |  |  | <0.001 |
| Median (Q1, Q3) | 3.74 (2.91, 4.76) | 3.23 (2.71, 4.18) | 3.47 (2.82, 4.38) | 3.74 (2.96, 4.76) | 4.48 (3.68, 6.50) |  |
| Min. - Max. | 0.44 - 20.71 | 0.44 - 12.39 | 0.99 - 17.30 | 1.14 - 13.90 | 1.71 - 20.71 |  |
| **Percent of Dendritic Cells** |  |  |  |  |  | 0.259 |
| Median (Q1, Q3) | 0 (0, 0) | 0 (0, 0) | 0 (0, 0) | 0 (0, 0) | 0 (0, 0) |  |
| Min. - Max. | 0 - 0.5198 | 0 - 0.5180 | 0 - 0.5198 | 0 - 0 | 0 - 0 |  |
| **Percent of M1 Macrophages** |  |  |  |  |  | <0.001 |
| Median (Q1, Q3) | 5.6 (3.3, 9.1) | 3.8 (2.3, 5.1) | 4.3 (3.1, 7.1) | 6.2 (4.2, 9.1) | 9.0 (6.3, 12.2) |  |
| Min. - Max. | 0 - 32.3 | 0 - 23.1 | 0.5 - 17.8 | 0.5 - 25.2 | 0.6 - 32.3 |  |
| **Percent of M2 Macrophages** |  |  |  |  |  | <0.001 |
| Median (Q1, Q3) | 5.50 (3.81, 7.59) | 4.63 (3.25, 6.38) | 5.13 (3.84, 6.81) | 5.52 (3.65, 7.45) | 6.79 (5.08, 8.57) |  |
| Min. - Max. | 0 - 20.47 | 0.45 - 16.21 | 0 - 14.18 | 0.05 - 18.19 | 0 - 20.47 |  |
| **Percent of Monocytes** |  |  |  |  |  | 0.002 |
| Median (Q1, Q3) | 0 (0, 0) | 0 (0, 0) | 0 (0, 0) | 0 (0, 0) | 0 (0, 0) |  |
| Min. - Max. | 0 - 7.87 | 0 - 7.72 | 0 - 7.87 | 0 - 6.30 | 0 - 2.99 |  |
| **Percent of NK Cells** |  |  |  |  |  | <0.001 |
| Median (Q1, Q3) | 2.74 (2.13, 3.45) | 2.42 (1.89, 3.05) | 2.54 (2.03, 3.16) | 2.85 (2.32, 3.59) | 3.10 (2.59, 3.92) |  |
| Min. - Max. | 0.28 - 9.09 | 0.59 - 9.09 | 0.28 - 6.13 | 1.15 - 5.98 | 1.21 - 7.43 |  |
| **Percent of Neutrophils** |  |  |  |  |  | 0.007 |
| Median (Q1, Q3) | 13 (9, 18) | 14 (9, 20) | 15 (10, 19) | 13 (9, 18) | 12 (9, 16) |  |
| Min. - Max. | 0 - 65 | 0 - 35 | 1 - 39 | 1 - 43 | 2 - 65 |  |
| **Percent of CD4 T Cells** |  |  |  |  |  | 0.145 |
| Median (Q1, Q3) | 0 (0, 0) | 0 (0, 0) | 0 (0, 0) | 0 (0, 0) | 0 (0, 0) |  |
| Min. - Max. | 0 - 26.70 | 0 - 7.90 | 0 - 8.11 | 0 - 18.79 | 0 - 26.70 |  |
| **Percent of CD8 T Cells** |  |  |  |  |  | <0.001 |
| Median (Q1, Q3) | 0.63 (0, 2.53) | 0 (0, 0.18) | 0.22 (0, 0.69) | 1.01 (0.38, 2.00) | 4.71 (2.98, 7.81) |  |
| Min. - Max. | 0 - 23.06 | 0 - 2.22 | 0 - 3.79 | 0 - 5.67 | 0 - 23.06 |  |
| **Percent of Tregs** |  |  |  |  |  | <0.001 |
| Median (Q1, Q3) | 2.83 (1.78, 4.21) | 1.78 (1.33, 2.58) | 2.36 (1.60, 3.27) | 3.14 (2.20, 4.07) | 4.62 (3.49, 5.86) |  |
| Min. - Max. | 0.13 - 15.29 | 0.13 - 6.75 | 0.25 - 7.64 | 0.29 - 10.76 | 0.68 - 15.29 |  |
| **Percent of Other Cells** |  |  |  |  |  | <0.001 |
| Median (Q1, Q3) | 61 (54, 67) | 66 (62, 71) | 63 (59, 69) | 62 (57, 66) | 51 (43, 58) |  |
| Min. - Max. | 8 - 90 | 51 - 81 | 35 - 80 | 30 - 90 | 8 - 71 |  |

**Supplement Table 4: Somatic DNA Alterations Stratified by LAG-3 RNA Expression**

|  | **Overall** | **Stratified by LAG-3 Expression** | | | | | **q-value*^1^*** |
| --- | --- | --- | --- | --- | --- | --- | --- |
|  |  | **<1.98** | **1.98-2.66** | **2.67-3.66** | **3.67+** | **P-value** |  |
| **Number of Patients** | N=566 | N=142 | N=141 | N=141 | N=142 |  |  |
| **VHL, N (%)** | 371 (66%) | 80 (56%) | 89 (63%) | 103 (73%) | 99 (70%) | 0.016 | 0.075 |
| **PBRM1, N (%)** | 211 (37%) | 58 (41%) | 50 (35%) | 55 (39%) | 48 (34%) | 0.596 | >0.9 |
| **SETD2, N (%)** | 144 (25%) | 35 (25%) | 38 (27%) | 40 (28%) | 31 (22%) | 0.609 | >0.9 |
| **TP53, N (%)** | 88 (16%) | 26 (18%) | 20 (14%) | 20 (14%) | 22 (15%) | 0.747 | >0.9 |
| **CDKN2A, N (%)** | 86 (15%) | 20 (14%) | 24 (17%) | 18 (13%) | 24 (17%) | 0.690 | >0.9 |
| **BAP1, N (%)** | 83 (15%) | 11 (7.7%) | 17 (12%) | 20 (14%) | 35 (25%) | <0.001 | 0.008 |
| **CDKN2B, N (%)** | 82 (14%) | 21 (15%) | 23 (16%) | 15 (11%) | 23 (16%) | 0.492 | >0.9 |
| **TERT, N (%)** | 66 (12%) | 12 (8.5%) | 16 (11%) | 12 (8.5%) | 26 (18%) | 0.031 | 0.11 |
| **KDM5C, N (%)** | 62 (11%) | 15 (11%) | 16 (11%) | 16 (11%) | 15 (11%) | 0.993 | >0.9 |
| **PTEN, N (%)** | 54 (9.5%) | 16 (11%) | 12 (8.5%) | 13 (9.2%) | 13 (9.2%) | 0.872 | >0.9 |
| **MTAP, N (%)** | 42 (7.4%) | 9 (6.3%) | 13 (9.2%) | 12 (8.5%) | 8 (5.6%) | 0.613 | >0.9 |
| **TSC1, N (%)** | 32 (5.7%) | 9 (6.3%) | 6 (4.3%) | 9 (6.4%) | 8 (5.6%) | 0.854 | >0.9 |
| **ARID1A, N (%)** | 32 (5.7%) | 7 (4.9%) | 8 (5.7%) | 9 (6.4%) | 8 (5.6%) | 0.964 | >0.9 |
| **NF2, N (%)** | 30 (5.3%) | 3 (2.1%) | 4 (2.8%) | 7 (5.0%) | 16 (11%) | 0.002 | 0.015 |

*1* False discovery rate correction for multiple testing

**Supplement Table 5: DNA Assay Distribution**
